# Gabapentin, opioids, and the risk of opioid-related death: A population-based nested case–control study

**DOI:** 10.1371/journal.pmed.1002396

**Published:** 2017-10-03

**Authors:** Tara Gomes, David N. Juurlink, Tony Antoniou, Muhammad M. Mamdani, J. Michael Paterson, Wim van den Brink

**Affiliations:** 1 Li Ka Shing Knowledge Institute, St. Michael’s Hospital, Toronto, Ontario, Canada; 2 Institute for Clinical Evaluative Sciences, Toronto, Ontario, Canada; 3 Institute of Health Policy, Management and Evaluation, University of Toronto, Toronto, Ontario, Canada; 4 Leslie Dan Faculty of Pharmacy, University of Toronto, Toronto, Ontario, Canada; 5 Sunnybrook Research Institute, Toronto, Ontario, Canada; 6 Department of Medicine, University of Toronto, Toronto, Ontario, Canada; 7 Department of Family and Community Medicine, University of Toronto, Toronto, Ontario, Canada; 8 Department of Medicine, St. Michael’s Hospital, Toronto, Ontario, Canada; 9 Department of Family Medicine, McMaster University, Hamilton, Ontario, Canada; 10 Department of Psychiatry, Academic Medical Center, University of Amsterdam, Amsterdam, The Netherlands; Massachusetts General Hospital, UNITED STATES

## Abstract

**Background:**

Prescription opioid use is highly associated with risk of opioid-related death, with 1 of every 550 chronic opioid users dying within approximately 2.5 years of their first opioid prescription. Although gabapentin is widely perceived as safe, drug-induced respiratory depression has been described when gabapentin is used alone or in combination with other medications. Because gabapentin and opioids are both commonly prescribed for pain, the likelihood of co-prescription is high. However, no published studies have examined whether concomitant gabapentin therapy is associated with an increased risk of accidental opioid-related death in patients receiving opioids. The objective of this study was to investigate whether co-prescription of opioids and gabapentin is associated with an increased risk of accidental opioid-related mortality.

**Methods and findings:**

We conducted a population-based nested case–control study among opioid users who were residents of Ontario, Canada, between August 1, 1997, and December 31, 2013, using administrative databases. Cases, defined as opioid users who died of an opioid-related cause, were matched with up to 4 controls who also used opioids on age, sex, year of index date, history of chronic kidney disease, and a disease risk index. After matching, we included 1,256 cases and 4,619 controls. The primary exposure was concomitant gabapentin use in the 120 days preceding the index date. A secondary analysis characterized gabapentin dose as low (<900 mg daily), moderate (900 to 1,799 mg daily), or high (≥1,800 mg daily). A sensitivity analysis examined the effect of concomitant nonsteroidal anti-inflammatory drug (NSAID) use in the preceding 120 days. Overall, 12.3% of cases (155 of 1,256) and 6.8% of controls (313 of 4,619) were prescribed gabapentin in the prior 120 days. After multivariable adjustment, co-prescription of opioids and gabapentin was associated with a significantly increased odds of opioid-related death (odds ratio [OR] 1.99, 95% CI 1.61 to 2.47, *p <* 0.001; adjusted OR [aOR] 1.49, 95% CI 1.18 to 1.88, *p <* 0.001) compared to opioid prescription alone. In the dose–response analysis, moderate-dose (OR 2.05, 95% CI 1.46 to 2.87, *p <* 0.001; aOR 1.56, 95% CI 1.06 to 2.28, *p =* 0.024) and high-dose (OR 2.20, 95% CI 1.58 to 3.08, *p <* 0.001; aOR 1.58, 95% CI 1.09 to 2.27, *p =* 0.015) gabapentin use was associated with a nearly 60% increase in the odds of opioid-related death relative to no concomitant gabapentin use. As expected, we found no significant association between co-prescription of opioids and NSAIDs and opioid-related death (OR 1.11, 95% CI 0.98 to 1.27, *p =* 0.113; aOR 1.14, 95% CI 0.98 to 1.32, *p =* 0.083). In an exploratory analysis of patients at risk of combined opioid and gabapentin use, we found that 46.0% (45,173 of 98,288) of gabapentin users in calendar year 2013 received at least 1 concomitant prescription for an opioid. This study was limited to individuals eligible for public drug coverage in Ontario, we were only able to identify prescriptions reimbursed by the government and dispensed from retail pharmacies, and information on indication for gabapentin use was not available. Furthermore, as with all observational studies, confounding due to unmeasured variables is a potential source of bias.

**Conclusions:**

In this study we found that among patients receiving prescription opioids, concomitant treatment with gabapentin was associated with a substantial increase in the risk of opioid-related death. Clinicians should consider carefully whether to continue prescribing this combination of products and, when the combination is deemed necessary, should closely monitor their patients and adjust opioid dose accordingly. Future research should investigate whether a similar interaction exists between pregabalin and opioids.

## Abstract 2

In a matched case-control-study among patients in Ontario, Canada treated with opioid painkillers, Tara Gomes and colleagues investigate whether co-prescription of gabapentin is associated with an increased risk of accidental opioid-related death.

## Author summary

**Why was this study done?:**

- Gabapentin is a drug often used together with opioids to treat chronic pain, and both of these drugs have been shown to suppress breathing, which can be fatal.
- Concomitant opioid use can also increase the amount of gabapentin absorbed by the body, potentially leading to higher risks when these drugs are used together.
- Despite these potential risks, so far no studies have examined whether the concomitant use of gabapentin and opioids really increases the likelihood of accidental opioid-related death and whether this risk is dependent on gabapentin dose.
- If there is an important interaction between these drugs, this would be of high clinical importance given the common combined use of these drugs.

**What did the researchers do and find?:**

- We conducted a matched case–control study among people treated with opioid painkillers in Ontario, the most populous province of Canada.
- We determined concomitant gabapentin exposure among 1,256 individuals (cases) who died of an opioid-related cause and 4,619 matched controls.
- We found that concomitant gabapentin and opioid exposure was associated with a 49% higher risk of dying from an opioid overdose.

**What do these findings mean?:**

- Clinicians should take great caution when combining gabapentin and opioids.
- Patients treated with opioids and gabapentin should be closely monitored, and may need to have their doses adjusted to avoid potential drug overdose.

## Introduction

Prescription opioid overdoses are an ongoing public health concern across North America, contributing to more than 15,000 deaths in the United States in 2015 [1]. Most opioid-related deaths result from respiratory depression, and co-administration of central nervous system (CNS) depressants is an important and avoidable risk factor for death. Recent evidence showed that co-prescription of benzodiazepines with opioids increases the risk of overdose death nearly 4-fold [2], leading to the introduction of black box warnings on the packaging for both products in August 2016 [3]. Furthermore, prescription opioid use has been shown to be highly associated with future risk of opioid-related death, with 1 of every 550 chronic opioid users dying within approximately 2.5 years of their first opioid prescription [4].

Gabapentin is an anticonvulsant commonly used as an adjunct for the treatment of chronic pain [5]. Although gabapentin is widely perceived as safe [5,6], drug-induced respiratory depression has been described when gabapentin is used alone or in combination with other medications [7–10]. Indeed, the product monograph was amended in 2014 to warn about possible respiratory depression when combined with opioids [11]. Potential risk factors for gabapentin-related respiratory depression include advancing age, renal insufficiency, chronic lung disease, and dose. The role of dose is particularly important in light of data indicating a 44% increase in systemic gabapentin exposure following its administration with morphine [8–10,12,13], likely reflecting increased drug absorption from lowered intestinal motility [12].

Because gabapentin and opioids are both commonly prescribed for pain, the likelihood of co-prescription is high [14–17]. However, to our knowledge, no published studies have examined whether concomitant gabapentin therapy is associated with an increased risk of accidental opioid-related death in patients receiving opioids. We investigated this question in a cohort of adults receiving prescription opioids in Ontario, Canada. We hypothesized that individuals co-prescribed opioids and gabapentin would have a higher risk of opioid-related death, and that there would be a dose–response gradient with increasing gabapentin dose.

## Methods

### Setting

We conducted a population-based nested case–control study of adults dispensed opioid analgesics under the Ontario Public Drug Programs between August 1, 1997, and December 31, 2013. All residents of Ontario receive publicly funded physician and hospital care. Public drug coverage for prescription medications is provided to individuals aged 65 years and older, as well as those who are unemployed, are receiving disability benefits, have high drug costs relative to their net household income, are receiving home care services, or reside in a long-term care home. The study protocol (S1 Text) was approved by the research ethics board of Sunnybrook Health Sciences Centre, Toronto, Ontario.

### Data sources

We used the Ontario Drug Benefit (ODB) database to identify prescription medications dispensed to eligible residents of Ontario over the study period, and the Ontario Health Insurance Plan database to identify services rendered by physicians. We used the Canadian Institute for Health Information’s Discharge Abstract Database and National Ambulatory Care Reporting System to identify diagnoses and procedures provided during hospital admissions and emergency department visits, respectively. We used the Registered Persons Database, which contains information on every Ontarian ever issued a health card, to identify demographic information. We used the Ontario Cancer Registry and the Ontario Diabetes Database (ODD) to capture diagnoses of cancer and diabetes, respectively. The ODD is a database constructed using administrative claims data that was shown to have a sensitivity of 86%, specificity of 97%, and positive predictive value of 80% against primary care physician diagnoses as the external criterion in a validation study [18]. Similarly, we used an algorithm in our hospitalization and physician claims data to define chronic kidney disease that has been shown in a validation study to have a specificity of 97% against laboratory measures of serum creatinine as the external criterion [19]. Finally, we abstracted detailed information on confirmed opioid-related deaths from the Office of the Chief Coroner of Ontario using methods described previously [20]. In Ontario, all sudden or unexpected deaths are investigated by a medical coroner to determine cause and manner of death. This rigorous investigation incorporates interviews as well as autopsy findings and postmortem toxicology to confirm cause of death. Our data capture all cases in which the findings of this investigation confirmed that opioids contributed to death, either alone or in combination with another drug or alcohol. Therefore, overdose deaths due to opioids alone or due to the combined sedating effects of opioids and gabapentin would be identified in this database. These data are used regularly to study opioid overdose deaths in Ontario [4,21–23]. All datasets were linked using unique, encoded identifiers, and were analyzed at the Institute for Clinical Evaluative Sciences (ICES; https://www.ices.on.ca).

### Identification of patients and outcomes

We identified a cohort of ODB-eligible individuals aged 15 to 105 years who were treated with at least 1 opioid prescription over the study period, including oral formulations of morphine, codeine, oxycodone, meperidine, and hydromorphone, as well as transdermal fentanyl patches. We excluded prescriptions for rarely used opioids (such as pentazocine or anileridine), parenteral or intranasal opioid formulations, and methadone, which in Ontario is principally used to treat opioid use disorders.

We defined cases as individuals from within this cohort who died from an opioid-related cause over the study period, and excluded opioid overdoses deemed to be suicides or homicides by the investigating coroner. We defined the index date as the date of death. The index date for potential controls was randomly assigned according to the distribution of index dates for included cases. We excluded individuals with invalid identifiers and those with a prior diagnosis of cancer or evidence of palliative care in the 6 months preceding the index date. Consequently, our analyses were limited to patients receiving opioids for non-cancer pain. We required that all study patients have at least 1 opioid prescription overlapping with their index date, and at least 6 months of continuous eligibility for public drug benefits prior to their index date.

To increase the comparability of cases and controls, we utilized a disease risk index to generate predicted probabilities of dying of an opioid-related death, using multiple demographic characteristics, medical problems, and psychiatric disorders, as described previously [22,24]. Details of the components of this risk index can be found in S1 Table. We used incidence density sampling to match each case with up to 4 controls on their disease risk index (within 0.2 standard deviations), age (within 5 years), sex, year of index date (within 1 year), and history of chronic kidney disease (prior 5 years). When a full number of matches could not be found, we matched as many controls as possible to each case, and any available controls were analyzed.

### Exposure definition

We defined recent concomitant gabapentin exposure on the basis of at least 1 prescription in the 120 days preceding each individual’s index date. In a secondary dose–response analysis, we stratified the gabapentin dose into low dose (<900 mg daily), moderate dose (900 to 1,799 mg daily), and high dose (≥1,800 mg daily) to align with dose ranges suggested in the product monograph (effective dose range 900 to 1,800 mg/day). Two post hoc sensitivity analyses were conducted. In the first, we further stratified high-dose gabapentin use into high dose (1,800 to 2,499 mg daily) and very high dose (≥2,500 mg daily) to investigate the association between opioid-related death and higher gabapentin doses. In the second post hoc sensitivity analysis, we defined gabapentin exposure on the basis of a gabapentin prescription where the days’ supply overlapped the index date. To test the specificity of our findings, we conducted a prespecified sensitivity analysis in which we examined recent concomitant exposure to nonsteroidal anti-inflammatory drugs (NSAIDs) in the prior 120 days because NSAIDs are not expected to independently increase the risk of opioid-related death among patients receiving opioids.

### Patient characteristics

We ascertained the average daily dose of opioids dispensed to each individual in the cohort by identifying all opioid prescriptions overlapping their index date. The daily dose of each prescription was calculated and converted into approximate milligrams of morphine or equivalent (morphine milligram equivalent [MME]) using morphine equivalence ratios defined by the Canadian National Opioid Use Guideline Group [25]. Opioid dose was grouped into categories as follows: <20 MME, 20 to 49 MME, 50 to 99 MME, 100 to 199 MME, and ≥200 MME.

We also identified a number of other patient characteristics, including sociodemographic characteristics (age, sex, urban/rural location of residence, income quintile), past medication use, Charlson Comorbidity Index (0, 1, 2 or more), history of alcohol use disorder, and health services utilization (S1 Table).

### Statistical analysis

We summarized patient characteristics using descriptive statistics, and used standardized differences to compare cases and controls. Standardized differences are often used instead of *p*-values when studying large cohorts, and values > 0.1 are generally considered to represent a meaningful difference [26]. Missing data for any covariates in the analysis were reported as separate categories. In accordance with ICES privacy policies, whenever a cell count was ≤ 5, the cell count was censored to avoid potential re-identification of personal health information.

We used conditional logistic regression to compare the odds of dying of opioid-related causes among opioid recipients co-prescribed gabapentin with the odds among those prescribed opioids alone. We estimated odds ratios (ORs) and 95% CIs for all comparisons, and adjusted all models for a number of important covariates, including opioid dose, age, medication use in the prior 120 days (pregabalin, selective serotonin reuptake inhibitor [SSRI] antidepressants, other antidepressants, benzodiazepines, other psychotropic drugs/CNS depressants, and methadone/buprenorphine, each considered separately), the number of drugs dispensed in the past 6 months, receipt of a long-acting opioid formulation during the exposure window, diagnosis of alcohol use disorder in the prior 3 years, Charlson Comorbidity Index, chronic lung disease, diabetes, number of opioid prescribers in the past 6 months, and number of pharmacies from which each person was dispensed opioids in the past 6 months. In our sensitivity analysis of concomitant NSAID use, we also adjusted the model for gabapentin use in the past 120 days.

To estimate the potential number of people at risk of combined opioid and gabapentin use, we created a cohort of gabapentin users in the last year of our study (calendar year 2013) and defined a period of ongoing gabapentin use on the basis of a prescription refill within 150% of the days’ supply of the prior prescription. We estimated the prevalence of co-prescription of gabapentin and opioids by identifying all individuals in this cohort who received at least 1 prescription for an opioid during the period of ongoing gabapentin use.

All analyses used a type I error rate of 0.05 as the threshold for statistical significance and were performed using SAS statistical software (version 9.4; SAS Institute, Cary, North Carolina).

The reporting of this study is in accordance with the REporting of studies Conducted using Observational Routinely collected health Data (RECORD) statement (S1 RECORD Checklist) [27].

## Results

**Fig 1:**
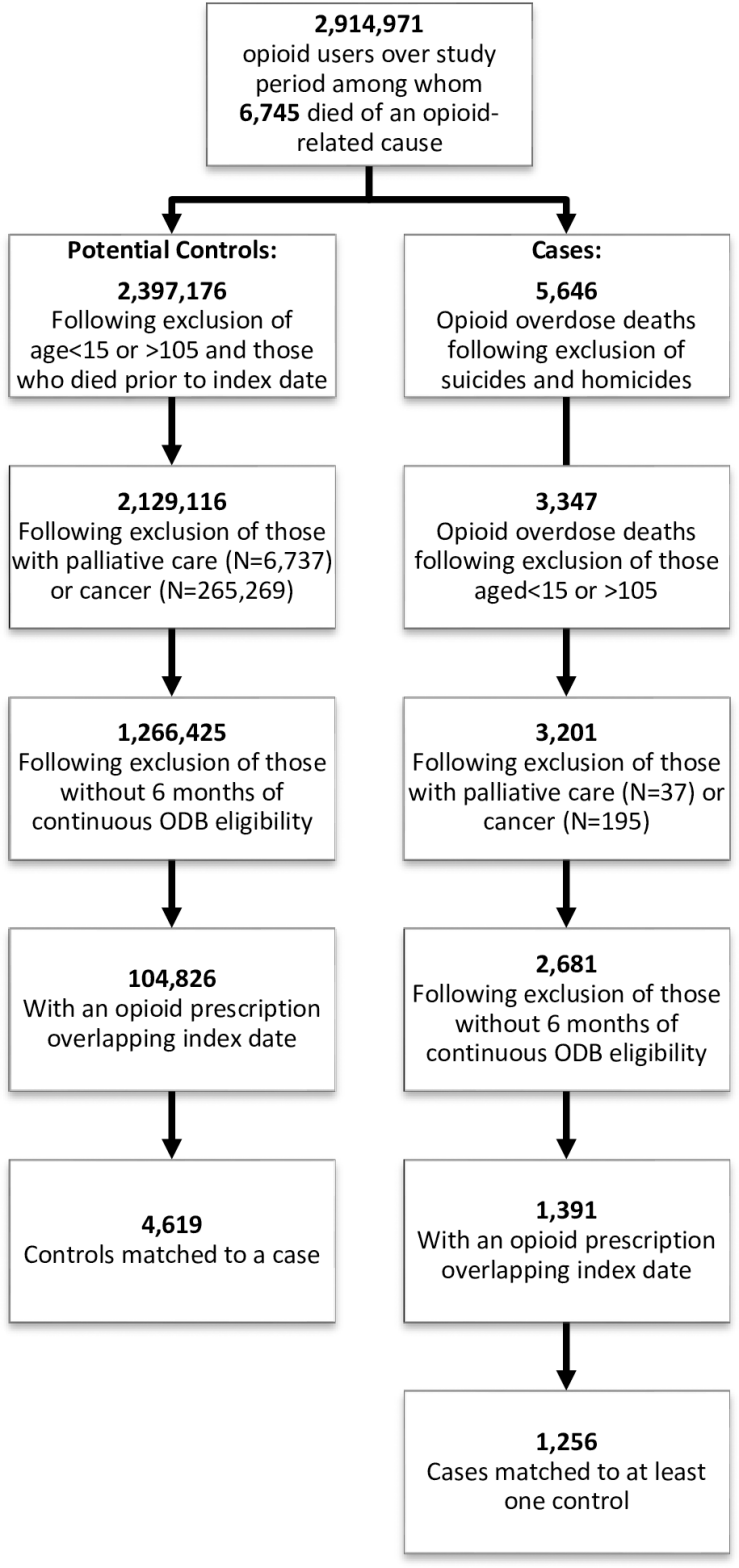
Inclusion/exclusion criteria applied to cases and controls. Details of cohort development, including identification of cases and potential controls, as well as inclusion and exclusion criteria applied prior to matching. ODB, Ontario Drug Benefit.

We identified 2,914,971 ODB-eligible individuals who received a prescription opioid over the study period; of these, 1,391 potential cases met our inclusion criteria (Fig 1), and 1,256 (90.3%) of these were matched to at least 1 control, leading to a total of 4,619 controls included in our study. All cases had prescription opioids found on postmortem toxicology, and 24 (1.9%) also had heroin present in their system at the time of death. Of those with heroin involved in their death, fewer than 6 had been exposed to a prescription for gabapentin in the prior 120 days.

Baseline characteristics of cases and controls are presented in Table 1. The majority of cases (94.5%) and controls (94.2%) were aged less than 65 years, and over 40% (42.1% of cases and 43.8% of controls) were in the lowest income quintile. As expected, cases tended to receive higher opioid doses, were more likely to have received a long-acting opioid during the exposure window, and were more likely to have recent exposure to antidepressants, benzodiazepines, and other CNS depressants. Cases had more visits to physicians and a greater number of prescriptions in the past 6 months.

**Table 1:** Baseline characteristics of individuals who died of an opioid overdose (cases) and matched controls.

Variable	Cases (*N =* 1,256)	Controls (*N =* 4,619)	Standardized Difference
***Demographic characteristics***			
**Age ≥ 65 years**	69 (5.5%)	266 (5.8%)	0.01
**Age, years (mean ± SD)**	47.5 ± 10.0	47.8 ± 9.9	0.03
Age < 65 years	46.4 ± 8.2	46.1 ± 8.3	0.03
Age ≥ 65 years	70.8 ± 6.2	70.7 ± 6.2	0.01
**Male**	716 (57.0%)	2,619 (56.7%)	0.01
**Income quintile**			
1	529 (42.1%)	2,025 (43.8%)	0.04
2	285 (22.7%)	1,047 (22.7%)	0.00
3	202 (16.1%)	699 (15.1%)	0.03
4	132 (10.5%)	477 (10.3%)	0.01
5	100 (8.0%)	341 (7.4%)	0.02
Missing	8 (0.6%)	30 (0.6%)	0.00
**Location of residence**			
Rural	150 (11.9%)	548 (11.9%)	0.00
Urban	1,103 (87.8%)	4,062 (87.9%)	0.00
Missing	≤5 (0.2%)	9 (0.2%)	0.01
***Opioid dose***			
<20 MME daily	141 (11.2%)	1,162 (25.2%)	0.37
20–49 MME daily	228 (18.2%)	1,340 (29.0%)	0.26
50–99 MME daily	202 (16.1%)	680 (14.7%)	0.04
100–199 MME daily	189 (15.0%)	488 (10.6%)	0.13
≥200 MME daily	496 (39.5%)	949 (20.5%)	0.42
***Prior medication use***			
**Number of drugs used in past 6 months (median [IQR])**	11 (7–15)	9 (6–13)	0.30
**Past medication use (120 days)**			
Antidepressants—SSRIs	566 (45.1%)	1,690 (36.6%)	0.17
Antidepressants—other	622 (49.5%)	1,736 (37.6%)	0.24
Benzodiazepines	971 (77.3%)	2,604 (56.4%)	0.46
Other psychotropic drugs/CNS depressants	448 (35.7%)	1,190 (25.8%)	0.22
Methadone/buprenorphine	78 (6.2%)	212 (4.6%)	0.07
Pregabalin	12 (1.0%)	29 (0.6%)	0.04
**Long-acting opioid during exposure window**	784 (62.4%)	1,828 (39.6%)	0.47
***Patient comorbidity***			
**Charlson Comorbidity Index**			
No hospitalization	551 (43.9%)	2,529 (54.8%)	0.22
0	388 (30.9%)	1,219 (26.4%)	0.10
1	171 (13.6%)	447 (9.7%)	0.12
2 or more	146 (11.6%)	424 (9.2%)	0.08
**History of alcohol use disorder**	327 (26.0%)	1,074 (23.3%)	0.07
**Chronic kidney disease**	58 (4.6%)	173 (3.7%)	0.04
**Mental health diagnoses**			
Affective disorder	256 (20.4%)	902 (19.5%)	0.02
Anxiety/sleep disorders	1,027 (81.8%)	3,779 (81.8%)	0.00
Psychoses	180 (14.3%)	572 (12.4%)	0.06
Other mental health diagnoses	913 (72.7%)	3,280 (71.0%)	0.04
**Chronic lung disease**	294 (23.4%)	1,040 (22.5%)	0.02
**Diabetes**	203 (16.2%)	734 (15.9%)	0.01
***Health services utilization (median [IQR])***			
Visits to a physician in past year	42 (23–78)	36 (19–64)	0.19
Doctors prescribing opioids in past 6 months	1 (1–2)	1 (1–2)	0.18
Pharmacies dispensing opioids in past 6 months	1 (1–2)	1 (1–2)	0.22

Data are number (percent) unless otherwise indicated.

CNS, central nervous system; MME, morphine milligram equivalent; SSRI, selective serotonin reuptake inhibitor.

**Fig 2:**
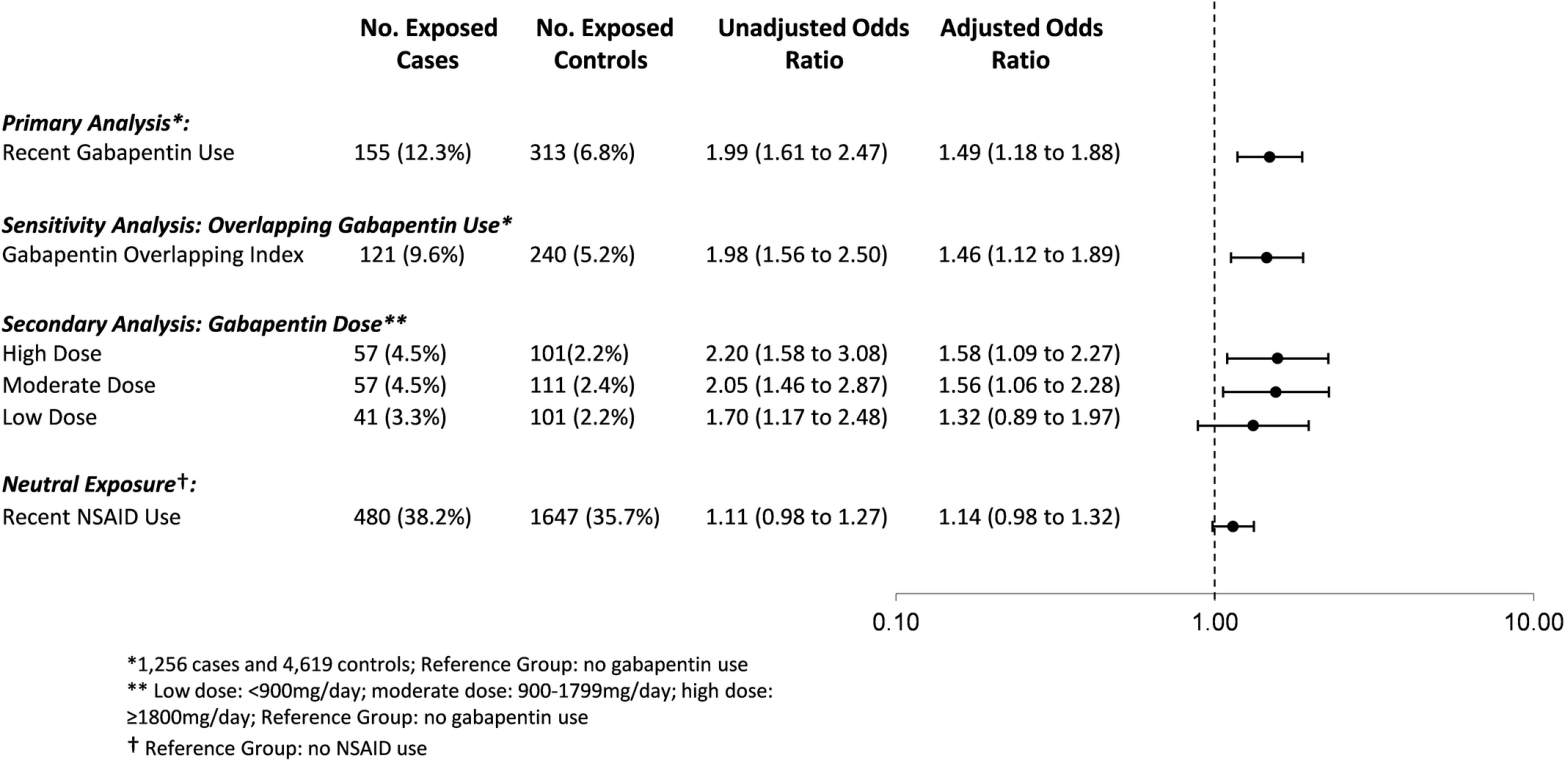
Association between co-prescription with gabapentin and opioids and opioid overdose. Association between co-prescription of gabapentin and opioids and opioid overdose, adjusted for age, opioid dose, medication use in the prior 120 days (pregabalin, SSRIs, other antidepressants, benzodiazepines, other psychotropic drugs/CNS depressants, long-acting opioid, methadone/buprenorphine), number of drugs dispensed in the past 6 months, alcohol use disorder, chronic lung disease, diabetes, Charlson Comorbidity Index, number of opioid prescribers in the past 6 months, and number of pharmacies dispensing opioids in the past 6 months. Neutral exposure model (NSAIDs) is also adjusted for gabapentin use in prior 120 days. NSAID, nonsteroidal anti-inflammatory drug.

Overall, 12.3% of cases (155 of 1,256) and 6.8% of controls (313 of 4,619) were prescribed gabapentin in the prior 120 days. In the primary analysis, we found that the odds of an opioid-related death was 49% higher among individuals recently exposed to gabapentin and opioids (adjusted OR [aOR] 1.49, 95% CI 1.18 to 1.88, *p <* 0.001) compared to those exposed to opioids alone, even after extensive adjustment for potential confounders, including opioid dose (Fig 2). In a sensitivity analysis considering only gabapentin prescriptions where the days’ supply overlapped the index date, the results were very similar (aOR 1.46, 95% CI 1.12 to 1.89, *p =* 0.005).

In our secondary analysis exploring the effect of gabapentin dose, we found that exposure to a moderate (900 to 1,799 mg daily) or high dose (1,800 mg daily or more) of gabapentin was associated with a nearly 60% increased odds of opioid-related death compared to exposure to opioids alone (aOR 1.56, 95% CI 1.06 to 2.28, *p =* 0.024, for moderate doses; aOR 1.58, 95% CI 1.09 to 2.27, *p =* 0.015, for high doses), whereas exposure to a low gabapentin dose was not significantly associated with an increased odds of opioid-related death (aOR 1.32, 95% CI 0.89 to 1.96, *p =* 0.174; Fig 2). In a post hoc sensitivity analysis, very high dose gabapentin use (2,500 mg daily or more) was associated with a nearly 2-fold increased odds of opioid-related death (aOR 1.83, 95% CI 1.04 to 3.22, *p =* 0.036).

Finally, in our sensitivity analysis to test the specificity of our findings, as expected, we found no significant association between recent exposure to concomitant NSAIDs and opioid-related death (aOR 1.14, 95% CI 0.98 to 1.32, *p =* 0.083).

In our exploratory analysis of patients at risk of combined opioid and gabapentin use, we found a total of 98,288 gabapentin users in calendar year 2013, of whom 45,173 (46.0%) received at least 1 concomitant prescription for an opioid. Furthermore, among our matched cases and controls, approximately 8% of opioid users had also recently received a prescription for gabapentin.

## Discussion

In this large study spanning more than 16 years, we found that approximately 8% of patients receiving opioids were co-prescribed gabapentin and that co-prescription was associated with a 50% increase in the risk of dying of opioid-related causes. A very high dose of co-prescribed gabapentin was associated with a near doubling of this risk. In contrast, and as expected, no such risk was observed among opioid recipients concomitantly prescribed NSAIDs. Our findings thus support the existence of a life-threatening drug–drug interaction between gabapentin and opioids in routine clinical practice.

The mechanism by which gabapentin may increase the risk of death in opioid users likely reflects both a pharmacodynamic and pharmacokinetic interaction [5]. More specifically, it likely reflects additive respiratory depression as well as increased gabapentin concentrations with concomitant opioid use [12]. A pharmacokinetic interaction most likely reflects increased gabapentin absorption, which occurs primarily in the upper small intestine [5]. Thus, opioid-induced slowing of gastrointestinal transit could prolong the time spent within this narrow absorption window and increase gabapentin bioavailability [5].

Our study has important implications for public health, particularly given the high degree of co-prescription. Almost 10% of patients treated with an opioid in our study also used gabapentin, while nearly half of patients treated with gabapentin were co-prescribed opioids. Similarly, studies from the United States and United Kingdom have estimated that between 15% and 22% of people with opioid use disorder are also misusing gabapentin [17]. Gabapentin is frequently used as an adjunct to opioids for neuropathic pain syndromes, but physicians may not be aware of the potential for respiratory depression with this drug; thus, increased awareness among patients and clinicians about the potential for a life-threatening interaction between these drugs is essential. When co-prescription is necessary, strategies for minimizing the sequelae of this interaction should be considered, including cautious dose titration, dose adjustment in the setting of co-morbid lung and kidney disease, and avoidance of other CNS depressants. In addition, patients treated with this combination should be instructed to seek medical attention immediately if symptoms of opioid overdose occur. Finally, because of pregabalin’s pharmacologic similarities to gabapentin, it is possible that pregabalin imparts a similar risk of overdose and death among opioid users, a hypothesis supported in part by cases of respiratory depression associated with this drug [28]. We were unable to test this hypothesis in our study due to the low prevalence of pregabalin prescribing over our study period (pregabalin only became available on the public drug program in 2013), but suggest that this is an important area of future research.

This study has several strengths, including its large size, the use of population-based coroner’s records to ascertain opioid-related deaths, and the specificity of the association of gabapentin co-prescription and opioid-related death (as evidenced by there being no such association between co-prescription of NSAIDs and opioid-related death). However, some limitations merit discussion. First, our study was limited to a population of individuals eligible for public drug coverage in Ontario, which includes the elderly (aged 65 and older) and younger individuals receiving social assistance. Approximately 95% of the cases and matched controls were younger than 65 years, which led the overall cohort to be generally of lower socioeconomic status (over 40% were in the lowest income quintile). Therefore, these findings may not be generalizable to the broader population of opioid users. Second, we were only able to identify prescriptions reimbursed by the government and dispensed from retail pharmacies. We could not determine drug adherence, whether individuals obtained additional drugs through cash payments or illicit purchases, or whether they were using their prescription opioids as prescribed. While it is possible that this could bias our analysis towards a significant finding if people concomitantly taking gabapentin and opioids were more likely to source opioids illicitly, our finding that heroin involvement in opioid-related deaths was highly concentrated among people not exposed to gabapentin suggests that this had no major influence on our findings. Third, the CIs in our dose–response analysis showed considerable overlap, and therefore the suggestion of a dose–response gradient should be interpreted with caution. Fourth, we were unable to determine the indication for gabapentin use, and so could not confirm whether these medications were being used to treat pain or for indications such as anxiety or depression. However, it is likely that the majority of gabapentin prescribing in this study was for a pain indication due to its concurrent use with opioids. Finally, despite matching on several demographic and clinical variables (including a disease risk index), our cases and controls differed on a number of measured covariates. Confounding by indication is also possible, particularly among patients receiving the highest doses of gabapentin or opioids, for whom severe pain could be a manifestation of serious underlying illness. However, we believe that confounding by indication did not have considerable influence on our findings for several reasons. First, we used a disease risk index to match cases to controls on a number of factors, including diagnoses commonly associated with pain. Second, because we adjusted for opioid dose in all of our analyses, our findings for gabapentin exposure are representative of the additional risk of opioid-related death beyond that which is explained by escalating opioid doses. Third, we adjusted our analyses for markers of severity of underlying disease, including comorbidity, medications, and health service use, and excluded individuals receiving palliative care and those with a prior cancer diagnosis. Finally, our tracer analysis found no association of NSAID co-prescription with opioid-related death, suggesting that underlying pain is not obfuscating our findings, and further mitigating concerns about confounding by indication.

### Conclusion

In this study we found that, among patients prescribed opioids, co-prescription of gabapentin was associated with a considerable increase in the risk of opioid-related death, particularly at higher doses. The clinical consequences of a potential drug–drug interaction are clear given the large number of people at risk of this fatal outcome. Clinicians should consider carefully whether to continue prescribing this combination of products and, when co-prescription is deemed necessary, should closely monitor their patients and adjust opioid dose accordingly. Future research should investigate whether a similar interaction exists between pregabalin and opioids.

## Supporting information

S1 RECORD Checklist RECORD statement and checklist. (DOCX)

S1 Table Components of disease risk index used to match cases to controls. (DOCX)

S1 Text Analytical plan. (DOCX)

## Abbreviations

aOR: adjusted odds ratio
CNS: central nervous system
ICES: Institute for Clinical Evaluative Sciences
NSAID: nonsteroidal anti-inflammatory drug
ODB: Ontario Drug Benefit
OR: odds ratio
SSRI: selective serotonin reuptake inhibitor
