# Supplementary material for: Gabapentin, opioids, and the risk of opioid-related death: A population-based nested case–control study
Source: PLoS Med. 2017 Oct 3;14(10):e1002396. doi: 10.1371/journal.pmed.1002396 (PMC5626029; doi:10.1371/journal.pmed.1002396)
Supplement: S1 Table — (DOCX) [file pmed.1002396.s002.docx]

**S1 Table. Components of Disease Risk Index used to Match Cases to Controls**

| **Variable** | **ICD9 Diagnosis Code** | **ICD10 Diagnosis Code** | **OHIP Diagnosis Code** |
| --- | --- | --- | --- |
| ***Demographic Characteristics (measured on index date)*** | | | |
| Age |  |  |  |
| Gender |  |  |  |
| Estimated residential income quintile | Neighbourhood Income Quintile based on location of residence on index date | | |
| Residence in a long-term care facility | Based on LTC flag on most recent ODB prescription in past 1 year | | |
| Rurality of Principal Residence | Rural community defined as community with <=10,000 residents | | |
| ***Medical disorders (measured by presence in 3 years prior to index date)*** | | | |
| Acute myocardial infarction | 410 | I21 | 410, 413 |
| Alcohol abuse | V113, 291, 303.0, 303.9, 305.0, 357.5, 425.5, 535.3, 571.0, 571.1, 571.3, 790.3, 980.0 | F10, G31.2, G62.1, G72.1, I42.6, I70.0, K29.2, K70.1, K70.4, K70.9, K86.0, R78.0, T51.0, X65, Y15, Y91, Z50.1, Z71.4, Z86.40 | 291, 303 |
| Atherosclerotic disease | 414.0, 440 | I251, I70 | 440 |
| Chronic lung disease | 490.0, 491, 492.0, 494.0, 496.0 | J40-J44, J47 | 491, 492, 494, 496 |
| Dementia | 290, 331.0, 331.1, 331.2, 797 | F00, F01, F02.0, F02.1, F03, F05.1, G30, G31.0, G31.1, R54 | 290, 331, 797 |
| Diabetes mellitus | Defined as diagnosis date in Ontario Diabetes Database that precedes the index date | | |
| Dyslipidemia | 272.0-272.6, 272.9 | E78.0-E78.6, E78.9, E88.1, H02.6 | 272 |
| Gastrointestinal hemorrhage | 531.0, 531.2, 531.4,  531.6, 532.0, 532.2,  532.4, 532.6, 533.0,  533.2, 533.4, 533.6,  534.0, 534.2, 534.4,  534.6, 578.0, 578.1,  578.9 | K25.0, K25.2, K25.4, K25.6, K26.0, K26.2, K26.4, K26.6, K27.0, K27.2, K27.4, K27.6, K28.0, K28.2, K28.4, K28.6, K92.0, K92.1, K92.2 |  |
| Glaucoma | 365 | H40, H42 | 365 |
| Gout | 274 | M10 | 274 |
| Heart failure | 428 | I50 | 428 |
| Hypothyroidism | 244.1, 244.3, 244.8, 244.9 | E01.8, E02, E03.2, E03.3, E03.5, E03.8, E03.9, E89.0 | 244 |
| Injury other than poisoning | 8XX, 90-95 | S*  T00-T35 |  |
| Osteoarthritis | 715 | M15.0-M15.2, M15.4, M16-M19, M89.41-M89.43, M89.45-M89.46, M89.48 | 715 |
| Other coronary heart disease | 411.0, 412.0, 413.0, 414.0, 414.8, 414.9 | I20, I24.0, I24.8, I24.9, I25.1, I25.2, I25.5, I25.6, I25.8, I25.9 | 412 |
| Parkinson’s disease | 332.0-332.1 | G20, G21.1, G21.2, G21.3, G21.8, G21.9, G22 | 332 |
| Pneumonia | 480-486 | J10-J18 | 486 |
| Poisoning or drug toxicity | 96-98 | T36-T65 | 977 |
| Rheumatoid arthritis | 714.0-714.4, 714.8 | M05, M06, M08.0, M08.2-M08.4, M08.8, M08.9, M09, M12.0 | 714 |
| Seizure disorder | 780.3, 345 | R56.0, R56.8, G40, G41 | 345 |
| Sepsis | 038 | A40, A41 |  |
| Stroke | 430-438 | I60, I61, I62,  I63, I64, G45 | 432-436 |
| Urinary incontinence | 788.3 | N39.3, N39.4, R32 |  |
| ***Psychiatric disorders (measured by presence in 3 years prior to index date)*** | | | |
| Affective disorder | 296 | F30, F31, F32.2, F32.3, F32.8, F33, F34.8, F34.9, F38, F39 | 296 |
| Anxiety or sleep disorders | 300 | F32.0, F34.1, F40-F42, F44, F45.0-F45.2, F48, F68.0, F99 | 300 |
| Psychoses, agitation, and related disorders | 292, 293.0, 293.8, 294, 295, 297.1, 297.3, 297.8, 297.9, 299 | F02.2-F02.4, F02.8, F04, F05.0, F05.8, F05.9, F06.0-F06.6, F06.8, F06.9, F09, F11.0, F11.3-F11.9  F12.0, F12.3-F12.9  F13.0, F13.3-F13.9  F14.0, F14.3-F14.9  F15.0, F15.3-F15.9  F16.0, F16.3-F16.9  F17.0, F17.3-F17.9  F18.0, F18.3-F18.9  F19.0, F19.3-F19.9  F20-F22, F23.2, F24, F25, F53.1, F84 | 295, 297 |
| All other mental disorders | 301, 302.2-302.9, 304.0-304.6, 304.9, 305.1-305.7, 305.9, 306.4, 306.5, 306.8, 306.9, 307, 308.3, 308.9, 309.0, 309.2, 309.8, 310.1, 310.2, 310.8, 310.9, 311.0, 312.0-312.3, 312.8, 312.9, 313.0, 313.2, 313.3, 313.8, 313.9, 314.0, 314.2, 314.8, 314.9, 315, 316.0 | F06.7, F07,  F11.1, F11.2  F12.1, F12.2 F13.1, F13.2 F14.1, F14.2 F15.1, F15.2 F16.1, F16.2 F17.1, F17.2 F18.1, F18.2 F19.1, F19.2  F32.9, F43, F45.3, F45.4, F45.8, F45.9, F50, F51, F52, F53.0, F54-F59, F60-F66, F68.1, F68.8, F69, F80-F83, F88-F95, F98, G44.2 | 301, 302, 304,  305, 306-316 |
| ***Other Variables*** | | | |
| Suicide attempt in 3 years prior to index date | 95.0-95.9 | X60-X84 |  |
| Number of visits to a physician in the past 1 year prior to index date | Count only one OHIP claim per person per physician per day. | | |
| Care by a psychiatrist in 1 year prior to index date | Main Specialty in IPDB = “PSYCHIATRY” | | |
| Days in hospital during 1 year prior to index date | Defined as sum of total length of stay of all hospitalizations discharged in prior year | | |

**S1 Table 2: Codes for Chronic Alcoholism and CKD**

|  | **ICD9** | **ICD10** | **OHIP Dxcode** |
| --- | --- | --- | --- |
| Alcohol abuse | V113, 291, 303.0, 303.9, 305.0, 357.5, 425.5, 535.3, 571.0, 571.1, 571.3, 790.3, 980.0 | F10, G31.2, F62.1, G72.1, I42.6, I70.0, K29.2, K70.1, K70.4, K70.9, K86.0, R78.0, T51.0, X65, Y15, Y91, Z50.1, Z71.4, Z86.40 | 291, 303 |
| Chronic Kidney disease  *Validated by Fleet et al.BMC Nephrology, Spec >92%, Sensitivity 33% for detecting eGFR<45mL/min, NPV 98.1%^1^* | 250.3, 403.90, 404.90, 581.8, 583.8, 585, 586 | E10.2, E11.2, E13.2, E14.2, I12, I13, N08, N18, N19 | 403, 585 |

**References**

1. Fleet JL, Dixon SN, Shariff SZ, et al. Detecting chronic kidney disease in population-based administrative databases using an algorithm of hospital encounter and physician claim codes. *BMC Nephrol.* 2013;14:81.
