# Supplementary material for: Gabapentin, opioids, and the risk of opioid-related death: A population-based nested case–control study
Source: PLoS Med. 2017 Oct 3;14(10):e1002396. doi: 10.1371/journal.pmed.1002396 (PMC5626029; doi:10.1371/journal.pmed.1002396)
Supplement: S1 Text — (DOCX) [file pmed.1002396.s003.docx]

| Project InitiationThis Section must be Completed Prior to Project Dataset(s) Creation | | | | | |
| --- | --- | --- | --- | --- | --- |
| **Project Title:** | Concomitant use of Opioids and Gabapentin and Opioid-Related Death | | | | |
| **Project TRIM number:** | 2016 0760 239 000 | | | | |
| **Research Program:** | CDP | | | | |
| **Site:** | ICES Central | | | | |
| **Project Objectives:** | *Insert Project Objectives as listed in the approved ICES Project PIA* | | | | |
|  | To examine whether the co-prescription with gabapentin among a nest of opioid users increases the risk of dying of opioid-related causes. | | | | |
| **ICES Project PIA Initial Approval Date:** | *The ICES Employee or agent who is responsible for creating the Project Dataset(s) is responsible for ensuring there is an approved ICES Project PIA and verifying the date of approval prior to creating the Project Dataset(s)* | | | | |
|  | 2016-MAR-29 | | | | |
| **Principal Investigator (PI):** | Tara Gomes | | | | |
| **Check the applicable box if the PI is an ICES Student/Trainee** | ICES Student  ICES Fellow  ICES Post-Doctoral Trainee  Visiting Scholar | | | | |
| **Responsible ICES Scientist:** | *Name the Responsible ICES Scientist if the PI is not a Full Status ICES Scientist* | | | | |
|  |  | | | | |
| **Project Team Member(s) Responsible for Project Dataset Creation and/or Statistical Analysis and date joined (list all):** | *All person(s) (ICES Analyst, Appointed Analyst, Analytic Epidemiologist, PI, and/or Student) responsible for creating the Project Dataset(s) and/or statistical analysis on the Research Analytics Environment (RAE) and the date they joined the project must be recorded* | | | | |
|  | Tara Gomes | | | 2016-Feb-17 | |
| **Other ICES Project Team Members and date joined (list all):** | *All other Research Project Team Members (e.g., Research Administrative Assistants, Research Assistants, Project Managers, Epidemiologists) and the date they joined the project must be recorded* | | | | |
|  | Muhammad Mamdani  David Juurlink  Michael Paterson  Samantha Singh  Tony Antoniou  Wim van den Brink | | | 2016-Feb-17  2016-Feb-17  2016-Feb-17  2016-Feb-17  2016-Feb-17  2016-Feb-17 | |
| **Confirmation that DCP is consistent with Project Objectives:** | *The following individuals must confirm that the ICES Data provided for in this DCP is relevant (e.g., with respect to cohort, timeframe, and variables) and required to achieve the Project Objectives stated in the ICES Project PIA prior to initial Project Dataset creation: 1) PI; 2) Responsible ICES Scientist if the PI is not a Full Status ICES Scientist, or a second ICES Scientist or the Scientific Program Lead if the PI is creating both the DCP and the Project Dataset[s]; 3) ICES Research Practice Staff creating the DCP; and 4) ICES Analytic Staff (ICES Employee or agent responsible for creating the Project Dataset[s]). This may be delegated either verbally or via e-mail.* | | | | |
|  | ***Principal Investigator*** | |  | | yyyy-mon-dd |
|  | ***Responsible ICES Scientist or Second ICES Scientist/Lead*** | |  | yyyy-mon-dd | |
|  | ***ICES Research Practice Staff Creating the DCP*** | |  | yyyy-mon-dd | |
|  | ***ICES Analytic Staff*** | |  | yyyy-mon-dd | |
| **Designated ICES Research Practice Staff accountable for Project Documentation:** | *The person named (ICES staff) is accountable for ensuring that the approved ICES Project PIA, ICES Project PIA Amendments, and DCP are saved on the T Drive, ensuring ICES Project PIA Amendments are submitted as required, ensuring DCP Amendments are documented, and sharing the final DCP with the PI/Responsible ICES Scientist at project completion* | | | | |
|  | Samantha Singh | | | | |
| **DCP Creation Date and Author:** | *Date DCP was finalized prior to Project Dataset(s) creation* | *Name of person who created the DCP* | | | |
|  | ***Date*** | ***Name*** | | | |
|  | 2016-Feb-17 | Tara Gomes | | | |

| ICES DataThis Section must be Completed Prior to Project Dataset(s) Creation | |
| --- | --- |
| *The ICES Employee or agent who is responsible for creating the Project Dataset(s) must ensure that this list includes only data listed in the ICES Project PIA*  *Changes to this list after initial ICES Project PIA approval require an ICES Project PIA Amendment* | *Mandatory for all datasets that are available by individual year* |
| ***General Use Datasets – Health Services*** | ***Years (where applicable)*** |
| CIHI DAD | 1994-2013 |
| CIHI SDS | 1994-2013 |
| NACRS | 2000-2013 |
| ODB | 1994-2013 |
| OHIP | 1994-2013 |
| ***General Use Datasets – Care Providers*** |  |
| IPDB |  |
| See list |  |
| ***General Use Datasets – Population*** |  |
| RPDB |  |
| See list |  |
| ***General Use Datasets – Coding/Geography*** |  |
| DIN |  |
| See list |  |
| ***General Use Datasets - Facilities*** |  |
| See list |  |
| ***General Use Datasets - Other*** |  |
| ODD |  |
| See list |  |
| See list |  |
| ***Controlled Use Datasets*** |  |
| OCR |  |
| See list |  |
| ***Other Datasets*** |  |
| Ontario Coroner’s Database | 1997-2013 |

| Project Amendments and Reconciliation | | | |
| --- | --- | --- | --- |
| **ICES Project PIA Amendment History (add additional rows as needed):** | *Privacy approval date* | *Person who submitted amendment* | *Note that any changes to the list of ICES Data or Project Objectives require an ICES Project PIA Amendment* |
|  | ***Date*** | ***Name*** | ***Amendment*** |
|  | yyyy-mon-dd |  |  |
| **DCP Amendment History (add additional rows as needed):** | *Date DCP amended* | *Person who made the DCP amendment* | *Note that any DCP amendments involving changes to the list of ICES Data or Project Objectives require an ICES Project PIA Amendment* |
|  | ***Date*** | ***Name*** | ***Amendment*** |
|  | 2016-Mar-09 | Tara Gomes | Revised methods to clarify gabapentin dose categories |

| Project Cohort | | |
| --- | --- | --- |
| **Study Design** | Cohort study  Matched cohort study  Case-control study  Cross-sectional study  Other (specify): | |
| **Nest Cohort** | Nest of people who are actively using a study opioid prior to index date and who have been ODB eligible for at least 6 months. Must have been prescribed at least 1 study opioid during the study period.  This will be defined in the case/control definition section below | |
| **Estimated Size of Cohort**  **(if known)** |  | |
| **Exclusions (in order)** | *Step* | Description |
|  | 1 | Invalid IKN |
|  | 2 | Age <15 or >105 |
|  | 3 | Individuals with evidence of Palliative Care in past 6 months prior to index date (using OHIP feecodes A945, B998, C945, C882, C982, K023, W872, W882, W972, W982 and DAD patserv=58) |
|  | 4 | Individuals with cancer (using diagnosis date from the OCR) prior to index date |
|  | 5 | Rx for non-oral/transdermal patch opioids (since can’t convert to morphine equivalents) and Rx for Methadone or Opioids rarely used that overlap index date |
|  | 6 | Individuals not eligible for ODB |
|  | 7 | Opioid-related deaths deemed to be suicides or homicides |

| Project Time Frame Definitions | | |
| --- | --- | --- |
| Look-back Window  Observation Window  (in which to look for outcomes)  **Index Event Date**  Accrual Window  Max Follow-up Date | |  |
| **Accrual Start/End Dates** | Aug 1 1997 – December 31, 2013 |  |

| Variable Definitions (add additional rows as needed) | | |
| --- | --- | --- |
| **Cases** | **Primary Outcome: Opioid-related mortality**   - Individuals from the nest cohort with opioid-related mortality identified using abstracted coroner’s data. - Exclude cases who:   - Have no opioid prescriptions that overlap the index date   - Do not have at least 1 Rx for any drug in the 180 to 365 days prior to index date   - Have history of palliative care in 6 months prior to death   - Have cancer diagnosis prior to death   - Manner of Death = Suicide or Homicide |  |
| **Eligible Controls** | - Individuals taking opioids at some point over study period. - Randomly assign index date following same distribution of index dates of cases - Exclude controls who:   - Have no opioid prescriptions that overlap the index date   - Do not have at least 1 Rx for any drug in the 180 to 365 days prior to index date   - Have history of palliative care in 6 months prior to death   - Have cancer diagnosis prior to death   - Have outcome prior to assigned index date   Case to Control Ratio: Match 4 controls to each case   - Matching criteria:   - Risk Score (+/- 0.2 SD) (See Supplementary Appendix for components)   - Age (+/- 5 years and age<65 vs. 65+)   - Sex   - Index Year (+/- 1 year)   - History of Chronic Kidney Disease (past 5 years; DAD, OHIP) - When full number of matched controls cannot be obtained, the matching process is maintained, and any available controls are analyzed. - Cases without at least 1 matched control are excluded - Each potential control can only be selected once - Cases can act as controls prior to death |  |
| **Index date** | **Case**: Date of outcome  **Control**: Randomly assigned over study period based on same distribution as cases. |  |
| **Main Exposure** | **Gabapentin Exposure defined as:**  **Gabapentin:**   1. **Recent gabapentin use:** Prescription for gabapentin in the 120 days prior to index date 2. **No use:** No prescription for gabapentin in the 120 days prior to index date   **Secondary Analysis: Gabapentin Dose:**   1. Stratify gabapentin exposure into low (<900mg), moderate (900-1800mg) and high (>1800mg) dose by calculating daily dose on prescription closest to index date.   **Neutral Exposure: NSAID use:**   1. Identify those with NSAIDs in 120 days prior to index. |  |
| **Baseline Characteristics** | **Opioid Dose**   - Only consider opioid Rx that overlap the index date - Calculate Average Daily Dose for each Rx that overlaps index date, convert to MEQ (as defined below) and sum to get average daily dose on index date in MEQ. - Define total morphine equivalents (MEQ) (multiply number pills dispensed * strength * conversion factor). - Define average daily dose for each prescription as total mg ME / Days Supply - Categorize average daily dose into: 1-19, 20-49, 50-99, 100-199, 200+   **Demographic Variables defined at cohort entry:**   - Age at index (mean and median) - Sex Male (N, %) - Urban/Rural/Missing location of residence - Other drug use in past 120 days:   - - Antidepressants – SSRIs     - Antidepressants - Other     - Benzodiazepine     - Other psychotropic drugs and other CNS depressants     - Methadone/buprenorphine     - Pregabalin - Income quintile - Number drugs use in past 6 months year - Charlson score (based on 5 years of hospitalization data) - History of alcoholism (past 5 years) - No. Doctors prescribing opioids in past 6 months (based on presc_i on ODB claims) - No. Pharmacies that have dispensed opioids in past 6 months (based on ag_i on ODB claims) - Whether Long-acting opioid dispensed during exposure window:   - LA Opioid Rx that overlaps index date - Number of physician visits in the past 1 year (keep one record per person per physician per day), median, IQR |  |

| Analysis Plan and Dummy Tables (expand/modify as needed) | | |
| --- | --- | --- |
| **Descriptive Tables (insert or append dummy tables), e.g.:** | | |
| **Table 1. Baseline characteristics for cases and matched controls** | | |
| **Table 2. unadjusted and adjusted OR for relationship between pregabalin/gabapentin use and risk of opioid overdose** | | |
|  | | |
| **Statistical Model(s)** | | |
| **Type of model** | Conditional logistic regression | |
| **Primary independent variable** | Gabapentin exposure | |
| **Dependent variable** | 1. Opioid-related death | |
| **Covariates** | Any unbalanced in Table 1 using standardized differences >0.10 + Opioid Dose categories | |
|  | |  |

| Quality Assurance Activities | | | |
| --- | --- | --- | --- |
| **RAE Directory of SAS Programs** |  | | |
| **RAE Directory of Final Dataset(s)** | *The* *final analytic dataset for each cohort includes all the data required to create the baseline tables and run all the models. It should include all covariates for all models such as patient risk factors, hospital characteristics, physician characteristics, exposure measures (continuous, categorical) and outcomes. It should include covariates that were considered but didn’t make the final cut. This would permit an analyst to easily re-run the models in the future.* | | |
|  |  | | |
| **RAE README file available:** Yes No | | | |
| **Date results of quality assurance tools for final dataset shared with project team (where applicable):** | | |  |
|  | | **%assign** | yyyy-mon-dd |
|  | | **%evolution** | yyyy-mon-dd |
|  | | **%dinexplore** | yyyy-mon-dd |
|  | | **%track / %exclude** | yyyy-mon-dd |
|  | | **%codebook** | yyyy-mon-dd |
| **Additional comments:** | |  | |

**Appendix: Codes for Chronic Alcoholism and CKD**

|  | **ICD9** | **ICD10** | **OHIP Dxcode** |
| --- | --- | --- | --- |
| Alcohol abuse | V113, 291, 303.0, 303.9, 305.0, 357.5, 425.5, 535.3, 571.0, 571.1, 571.3, 790.3, 980.0 | F10, G31.2, F62.1, G72.1, I42.6, I70.0, K29.2, K70.1, K70.4, K70.9, K86.0, R78.0, T51.0, X65, Y15, Y91, Z50.1, Z71.4, Z86.40 | 291, 303 |
| Chronic Kidney disease  *Validated by Fleet et al.BMC Nephrology, Spec >92%, Sensitivity 33% for detecting eGFR<45mL/min, NPV 98.1%* | 250.3, 403.90, 404.90, 581.8, 583.8, 585, 586 | E10.2, E11.2, E13.2, E14.2, I12, I13, N08, N18, N19 | 403, 585 |
